# Ecdysone Receptor-based Singular Gene Switches for Regulated Transgene Expression in Cells and Adult Rodent Tissues

**DOI:** 10.1038/mtna.2016.74

**Published:** 2016-09-27

**Authors:** Seoghyun Lee, Kyung-Cheol Sohn, Dae-Kyoung Choi, Minho Won, Kyeong Ah Park, Sung-Kyu Ju, Kidong Kang, Young-Ki Bae, Gang Min Hur, Hyunju Ro

**Affiliations:** 1 Department of Biological Sciences, College of Bioscience and Biotechnology, Chungnam National University, Daejeon, Republic of Korea; 2 Department of Dermatology, College of Medicine, Chungnam National University, Daejeon, Republic of Korea; 3 Department of Pharmacology, College of Medicine, Chungnam National University, Daejeon, Republic of Korea; 4 Affiliated Research (and Development) Institute, Daejeon, Republic of Korea; 5 Comparative Biomedical Research Branch, Research Institute, National Cancer Center, Goyang, Republic of Korea

**Keywords:** adenovirus, controlled gene expression, ecdysone receptor (EcR)-based gene regulatory system, tetracycline, tebufenozide

## Abstract

Controlled gene expression is an indispensable technique in biomedical research. Here, we report a convenient, straightforward, and reliable way to induce expression of a gene of interest with negligible background expression compared to the most widely used tetracycline (Tet)-regulated system. Exploiting a *Drosophila* ecdysone receptor (EcR)-based gene regulatory system, we generated nonviral and adenoviral singular vectors designated as pEUI(+) and pENTR-EUI, respectively, which contain all the required elements to guarantee regulated transgene expression (GAL4-miniVP16-EcR, termed GvEcR hereafter, and 10 tandem repeats of an upstream activation sequence promoter followed by a multiple cloning site). Through the transient and stable transfection of mammalian cell lines with reporter genes, we validated that tebufenozide, an ecdysone agonist, reversibly induced gene expression, in a dose- and time-dependent manner, with negligible background expression. In addition, we created an adenovirus derived from the pENTR-EUI vector that readily infected not only cultured cells but also rodent tissues and was sensitive to tebufenozide treatment for regulated transgene expression. These results suggest that EcR-based singular gene regulatory switches would be convenient tools for the induction of gene expression in cells and tissues in a tightly controlled fashion.

## Introduction

Precise regulation of transgene expression under the control of an inducible promoter that responds to treatment with various small molecules or employment of stresses such as heat shock or hormones is an important technique^1,2,3^ for both basic biological researches to elucidate the function of genes of interest and for clinical studies to develop safer gene therapies. Inducible gene expression systems should fit several criteria, including specific directional induction, a dose- and time-dependent response to the specific inducer, very low leaky expression, abolition of gene expression by depleting the inducer, and extremely low toxicity of the inducer.^1,2,3^ Among the several gene inducible systems, the tetracycline (Tet) transactivator (Tet-On/Off) system (termed the Tet system henceforth) exploiting Tet or its derivatives such as doxycycline as an inducer has been widely used to induce transgene expression.^4,5,6^ Although the binary feature (driver and effector plasmids) of the Tet system was adopted as a reliable transgene induction method, it is cumbersome and time-consuming to generate transgene-incorporated stable cell lines because at least two rounds of selection processes are required. The other drawback of the binary feature of the commercially available Tet system is that it is difficult to achieve the even distribution of the separate two plasmids in target cells by transient cotransfection experiments,^3^ thus resulting in the inadvertent production of a heterogeneous population of cells equipped with either the driver or the effector plasmid at different levels, rendering the quantitative measurements of transgene induction or single-cell tracking difficult. In addition, the considerable level of leakiness of the Tet system hampers its broad applications in biological researches, which require precisely controlled gene expression. Accordingly, several approaches have sought to circumvent the limitations of the conventional Tet system by using a single vector equipped with all the required Tet system elements to overcome the possible clonal variations inevitably caused by two rounds of transfections for the establishment of stable cell lines.^7,8^ These approaches use a chimeric Tet driver fused with diverse regulatory domains including the mSin3-interacting domain of human Mad1 (ref. 8), a ligand-binding domain of a mutated glucocorticoid receptor,^9^ and a domain of ecdysone receptor (EcR) to reduce the basal level of expression of transgenes,^9^ or exploit Cre recombinase to dampen the initial leakiness of transgene induction.^10^ However, the sharing of a poly A signaling sequence by two transcriptional units that is able to cause transcriptional interference due to the inhibitory effect of one transcriptional activity on a second transcriptional process,^7,11,12^ the binary configurations of the dual regulatory systems,^9,10^ and the lack of initial repression of transgene expression^8^ still hamper the application of the engineered Tet systems in biological studies.

To overcome these shortcomings inherent to the Tet system, several other transgene regulatory modules have been developed including a *Drosophila melanogaster* EcR-based gene inducible system.^3,13,14,15,16,17,18^ The nuclear hormone receptor EcR and its heterodimeric partner ultraspiracle regulate morphological changes in insects.^3^ When bound to the EcR/ultraspiracle heterodimer, ecdysone induces binding of EcR to DNA regulatory elements.^3,13^ The vertebrate retinoid X receptor (RXR), an ortholog of insect ultraspiracle, can form a heterodimer with EcR to gain transcriptional activity.^17^ Vertebrates do not have ultraspiracle; therefore, EcR and RXR must be coexpressed in vertebrate cells or tissues to induce a gene of interest by ecdysone or its agonist. Endogenous RXR may interfere with the heterodimeric feature of the EcR-based gene induction level; therefore, Padidam *et al.*^19^ replaced the EcR DNA-binding and activation regions with heterologous GAL4 DNA-binding and VP16 activation domains fused to the ligand-binding region of EcR. This fusion protein does not bind to endogenous RXR and forms a homodimer to gain transcriptional potential.^19^ Recently, a chimeric construct comprised of a minimal VP16 activation domain with the GAL4 DNA-binding domain and modified EcR (GvEcR) was reported to form a homodimer in the cytosol in the absence of an ecdysone agonist, tebufenozide.^9,17^ In the presence of tebufenozide, GvEcR enters the nucleus and then binds to an upstream activation sequence (UAS), thereby acting as a transcriptional activator.

However, although the GvEcR/UAS-dependent nonviral gene induction switch was successfully applied and validated *in vivo*,^17^ the binary feature of the system which would render unequal expression of the two units, leading to inconsistent transgene induction by chemical ligands has hampered its broad application in physiological and pathophysiological studies. Moreover, a GvEcR-based adenoviral gene switch has not been developed. In an effort to develop more convenient packaging systems for use in mammalian cells or for *in vivo* delivery, we created novel GvEcR-based nonviral and adenoviral gene inducible cassettes with singular identities equipped with the complete regulatory units required for chemical induction of transcription of a target gene. Herein, we report that our novel GvEcR-based singular system had rapid and potent transgene induction capabilities in the presence of a wide range of tebufenozide concentrations, with exceptionally low leaky expression *in vitro* and *in vivo*. More importantly, we also confirmed that our inducible adenoviral system functions in a spatiotemporal manner to express transgenes in an *in vivo* skin model. Thus, the system acts as a tight on/off switch for regulating gene expression, which provides a convenient tool for physiological and pathophysiological studies.

## Results

### Comparison of the EcR-based singular gene switch with the singular Tet-On system in transiently transfected cells

**Figure 1:**
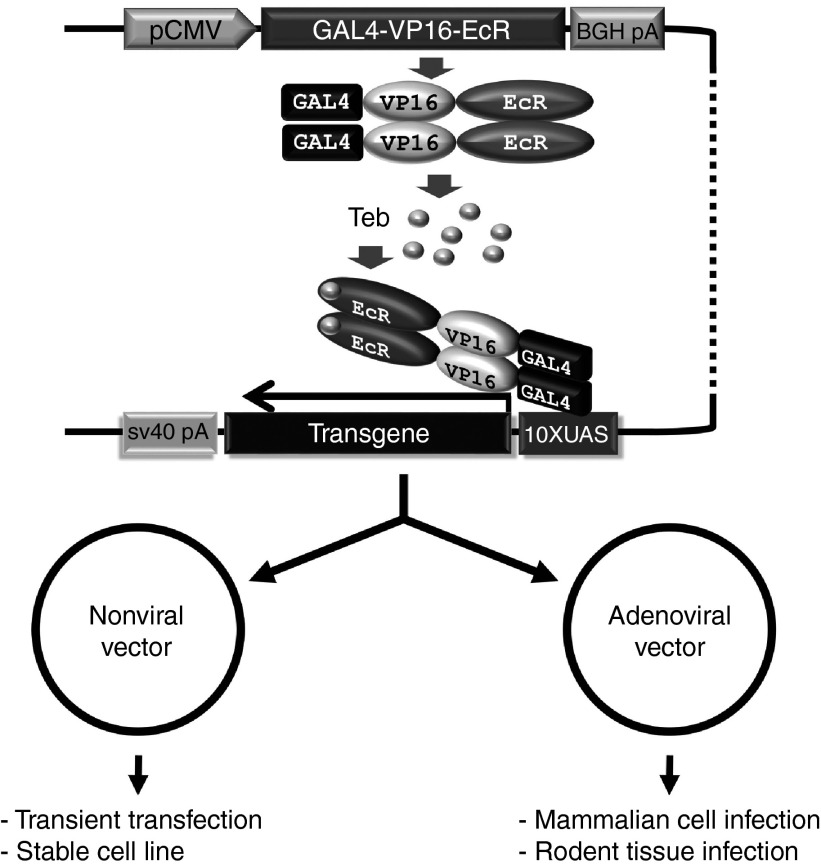
**A schematic illustration of the EcR-based singular transgene inducible switch**. The depicted Gal4-VP16-EcR chimeric transcriptional activator is constitutively produced under the control of a CMV promoter. The chimeric proteins remain in the cytosol in the absence of an ecdysone agonist, tebufenozide. Administration of tebufenozide triggers the translocation of Gal4-VP16-EcR from the cytosol to the nucleus, where it binds to 10×UAS and stimulates basal transcriptional machinery to initiate transcription of the downstream transgene. The entire transgene regulatable unit was transferred en bloc into a nonviral vector or an adenoviral vector. Abbreviations: Teb, tebufenozide; CMV, cytomegalovirus; EcR, ecdysone receptor; UAS, upstream activation sequence; 10×UAS, 10 tandem repeats of UAS.

**Figure 2:**
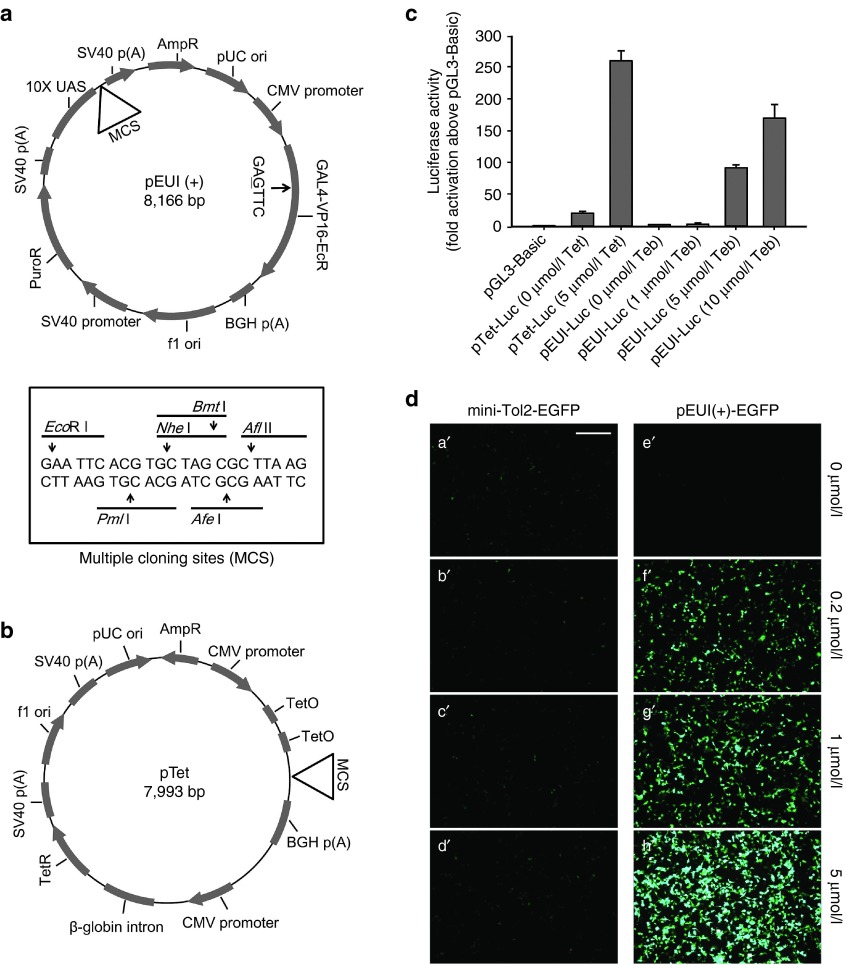
**An EcR-based singular gene switch induces robust transgene expression with low background expression.** (**a**) A schematic diagram of the pEUI(+) vector optimized for transgene expression under the control of a chemical inducer, tebufenozide. A single-nucleotide substitution of A to G at nucleotide position 4,783 was introduced to erase the *Eco*R I recognition sequence (arrow) without altering the amino acid sequence. The box shows the nucleotide sequences of the MCS of pEUI(+) with the restriction endonuclease recognition sites. (**b**) A schematic drawing of the singular pTet vector that responds to Tet. (**c**) Comparison of pEUI(+) and pTet vector capacities through the measurement of firefly Luc activity. Cells transfected with pEUI(+)-Luc show very low background expression (~1.7-fold increase versus pGL3-Basic) compared with pTet-Luc-transfected cells (~18.7-fold). pEUI(+)-Luc shows tebufenozide-dose-dependent transgene stimulation. Luc activity was normalized to cotransfected *Renilla* Luc activity. (**d**) The fluorescence intensity in pEUI(+)-EGFP-transfected cells increases in a tebufenozide-dose-dependent manner (**e'–h'**). The mini-Tol2-EGFP vector lacking a promoter was used for control transfection (**a'–d'**). HEK293T cells transfected with the indicated plasmid constructs were treated with tebufenozide (0–5 μmol/l) for 12 hours and then examined under a fluorescence microscope. Scale bar in a', 200 μm. EcR, ecdysone receptor; MCS, multiple cloning site; Tet, tetracycline.

To simplify the previously reported GvEcR-based transgene inducible system,^17^ we converted the binary feature of this system into a singular feature harboring all the elements required to induce transgene expression (**Figure 1**). The effector responding to the driver comprises 10 tandem repeats of UAS (10×UAS) next to an E1b minimal promoter and a downstream multiple cloning site (MCS) containing 5′- *Eco*R I, *Pml* I, *Nhe* I, *Bmt* I, *Afe* I, and *Afl* II -3′ recognition sequences followed by a SV40 poly(A) signal sequence (**Figure 2a**). For convenient cloning of a transgene into the MCS, we erased the *Eco*R I recognition site located between the Gal4 and VP16 domains of the original clone without altering the amino acid sequence (**Figure 2a**). Additionally, to facilitate the convenient selection of transfected cells, a SV40 promoter-driven puromycin *N*-acetyl-transferase (*PAC*) gene was placed between the driver and effector regions (**Figure 2a**). The newly designed vector, which was designated as pEUI(+) (GenBank accession no. KP123436; **Figure 2a**), was constructed primarily by ligation-independent cloning technology.^20,21^

To assess the activity and leakiness of pEUI(+), we first inserted a gene encoding firefly *luciferase* (*Luc*) into the *Eco*R I site in the MCS (pEUI(+)-Luc), transiently transfected the construct into HEK293T cells, treated the cells with various concentrations of tebufenozide for 12 hours, and then quantified the expression levels of the reporter using a dual-Luc reporter assay system (Promega, Madison, WI). The pGL3-Basic vector (Promega) harboring the *Luc* gene was used as a negative control. For comparison, we also generated a singular vector (pTet-Luc) for the Tet-dependent transgene inducible system by combining the pcDNA 6/TR inducer and the pcDNA 4/TO effecter with the *Luc* reporter gene (Invitrogen, Waltham, MA) (**Figure 2b**). Untreated pEUI(+)-Luc-transfected cells showed extremely low basal activity (~1.7-fold increase compared with pGL3-Basic, **Figure 2c**), as opposed to the more than 18.7-fold increase in untreated pTet-Luc-transfected cells. Addition of 5 μmol/l Tet increased the reporter activity in pTet transfectants by ~12.4-fold compared with the control (**Figure 2c**). Potent induction of transgene expression by the pTet system (increase of more than 233-fold compared with pGL3-Basic) was offset by its relatively high leaky expression. Subtraction of leaky expression from induced expression revealed that pEUI(+) is a more potent transgene induction system than pTet (47.5-fold versus 12.4-fold upon treatment with 5 μmol/l of the respective inducer). HEK293T cells transfected with pEUI(+) harboring the *EGFP* gene instead of *Luc* (termed pEUI(+)-EGFP) showed no discernible leaky expression when untreated and robust induction of expression by tebufenozide in a dose-dependent manner (**Figure 2d**). The vector encoding mini-Tol2-EGFP without any functional promoter^22,23^ was used as a transfection control. Mini-Tol2-EGFP-transfected cells did not respond to even 5 μmol/l tebufenozide treatment (**Figure 2d**), indicating tightly regulated transgene induction in the pEUI(+) gene switch cassette.

### The stably incorporated pEUI(+) gene switch in HEK293T cells shows tightly regulated transgene induction in a tebufenozide-dependent manner

**Figure 3:**
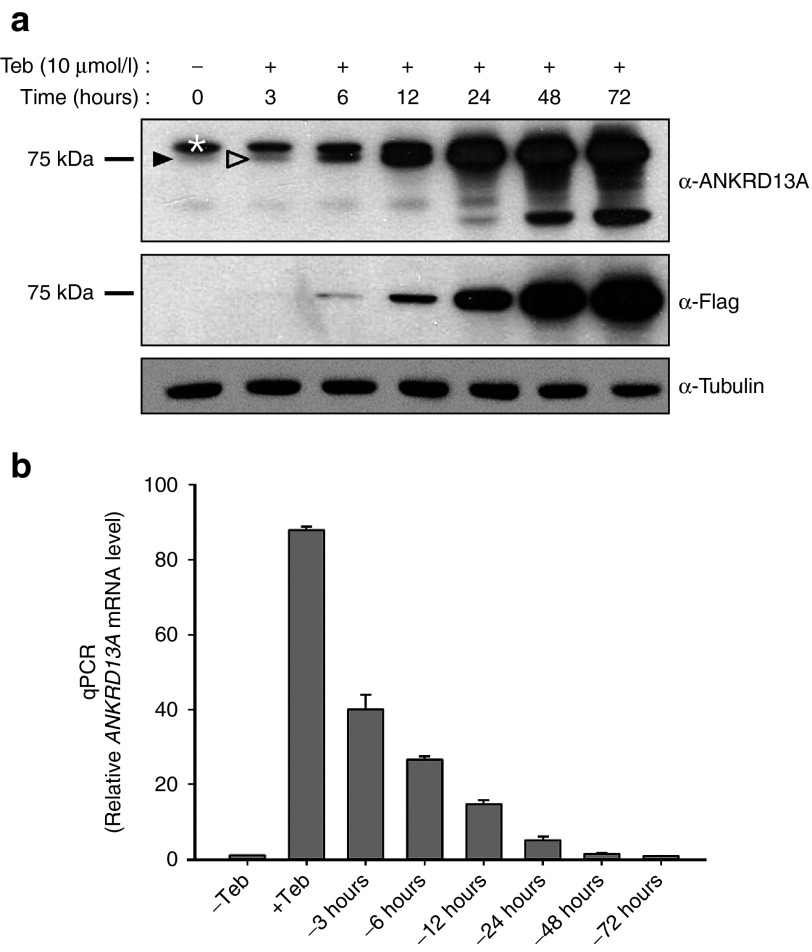
**HEK293 cells stably harboring pEUI(+)-ANKRD13A (#293-13A) show robust and reversible induction of ANKRD13A expression upon tebufenozide treatment**. (**a**) Induction of Flag epitope-tagged ANKRD13A expression is obvious in the #293-13A cell line after treatment with tebufenozide (10 μmol/l) for at least 3 hours (open arrowhead). ANKRD13A expression accumulates over time until 72 hours. Faint endogenous expression of ANKRD13A was detected with an anti-ANKRD13A antibody in a sample not treated with tebufenozide (dark arrowhead). Exogenous ANKRD13A expression was confirmed by immunoblotting using an anti-Flag antibody. Note that there is no detectable leaky expression of the transgene. The white asterisk indicates an unidentified cross-reactive species. (**b**) Comparison of the quantitative polymerase chain reaction (qPCR) yield based on triplicate analysis. #293-13A cells were treated with tebufenozide (10 μmol/l) for 24 hours, recultured in tebufenozide drop-out media for the indicated amount of time, and processed for cDNA synthesis. The relative amount of *ANKRD13A* transcripts was measured by PCR with ANKRD13A-specific primers. Teb, tebufenozide.

Ankyrin repeat domain 13A (ANKRD13A) belongs to a family of ankyrin repeat domain- and ubiquitin-interacting motif-containing proteins, which is implicated in a variety of cellular functions including ligand-mediated receptor endocytosis via protein-protein interactions.^24,25^ To elucidate the cellular roles of ANKRD13A, whose function remains obscure, we tried to generate a stable cell line harboring *ANKRD13A* under the control of GvEcR/UAS. We first inserted the Flag epitope-tagged *ANKRD13A* gene into the *Eco*R I site of pEUI(+) (termed pEUI(+)-ANKRD13A) and established a HEK293 cell line that stably harbors pEUI(+)-ANKRD13A (termed #293-13A hereafter, see **Supplementary Figure S1**). Subsequently, this stable cell line was stimulated with tebufenozide (10 μmol/l), lysed, and processed for western blot analysis using anti-ANKRD13A or anti-Flag M2 antibodies. ANKRD13A expression started to emerge at 3 hours after treatment with tebufenozide (10 μmol/l), and the transgene product accumulated over time (**Figure 3a**). Most importantly, we could not detect any background expression of the transgene even in a longer exposure (**Figure 3a**), again indicating tightly regulated transgene induction by pEUI(+). We are currently using the 293-13A cell line to study the detailed cellular and molecular functions of ANKRD13A.

To evaluate how long the effect of tebufenozide on the expression of target genes lasts, the #293-13A cell line was stimulated with 10 μmol/l tebufenozide for 24 hours, harvested, washed thoroughly several times with phosphate-buffered saline (PBS) to completely eliminate any remaining tebufenozide, reseeded onto new cell culture plates, cultured in tebufenozide-free growth media for the amount of time indicated in **Figure 3b**, and processed for total RNA extraction. Prepared cDNA was subjected to quantitative polymerase chain reaction (qPCR) with primer sets specific for *ANKRD13A* and *glyceraldehyde-3-phosphate dehydrogenase (GAPDH)*. The yield of *ANKRD13A* qPCR products was normalized to that of *GAPDH*. The transcription of *ANKRD13A* increased by 88-fold upon tebufenozide stimulation for 24 hours (**Figure 3b**), which gradually decreased upon removal of tebufenozide. Incubation of cells for 3 hours in tebufenozide-depleted media reduced expression of the transgene by 55%, and the transcription yield was decreased to basal levels at 72 hours after removal (**Figure 3b**), indicating the reversibility of transgene induction by the pEUI(+) system.

### The adenoviral singular gene switch is highly responsive to tebufenozide treatment in cells

**Figure 4:**
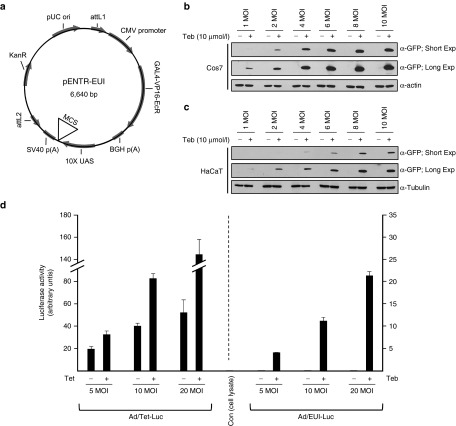
**An adenoviral singular gene switch induces tightly regulated transgene expression in cultured cell lines.** (**a**) A schematic diagram of the pENTR-EUI vector optimized for transgene expression under the control of tebufenozide treatment. The Gal4-VP16-EcR sequence information is identical to that in the pEUI(+) vector. The nucleotide sequence of the multiple cloning site (MCS) of pENTR-EUI is identical to that of pEUI(+).Cos7 cells (**b**) or HaCaT cells (**c**) were treated with purified adenoviruses encoding the *EGFP* reporter gene for 24 hours at a multiplicity of infection (MOI) of 1–10. After exchanging the culture medium, cells were grown for 24 hours with tebufenozide (10 μmol/l) before immunoblotting with an anti-GFP antibody. The endogenous level of α-actin (**b**) or α-tubulin (**c**) was measured by western blotting to validate the equal loading of each sample. Short and long exposures show the range of responsiveness of the adenoviral vector to tebufenozide. (**d**) Comparison of the capacities of the pENTR-EUI and pENTR-Tet (**Supplementary Figure S2**)-based adenoviral vectors through the measurement of firefly Luc activity. HaCaT cells were treated with the indicated adenoviral particles at an increasing MOI. Note that cells treated with Ad/EUI-Luc did not show any detectable background expression in any experimental conditions (2.18-, 0.83-, and 1.36-fold increase with a low, medium, and high dose of virus particles, respectively, versus control cell lysate). Leaky transgene expression was increased in Ad/Tet-Luc-infected cells in a virus dosage-dependent manner (1,661-, 3,399-, and 4,431-fold increase with a low, medium, and high dose of virus particles, respectively, versus control cell lysate). Teb, tebufenozide; Tet, tetracycline.

Adenoviral vectors have been widely used for clinical gene therapy^26,27^ because they can infect a variety of cell lines including nondividing cells and do not affect the integrity of the host genome due to the episomal state of the virus genome. To further facilitate use of the EcR-based singular gene switch in a wide range of cells and tissues, we adapted the adenoviral vector system. Using pENTR/D-TOPO (Invitrogen) as a vector backbone, we generated an amplifiable entry vector (GenBank accession no. KP123436; designated as pENTR-EUI, **Figure 4a**), which can deliver the entire regulatable gene expression units with an *EGFP* transgene subcloned in *Eco*R I of the MCS (**Figure 4a**) to a pAd/PL-DEST destination vector (Invitrogen) for adenovirus production. The infection ability and fidelity of regulated gene expression of the purified adenovirus was initially tested in Cos7 cells. Infected Cos7 cells were highly responsive to treatment with tebufenozide (10 μmol/l) in a multiplicity of infection (MOI)-dependent manner without any detectable background expression, even in the prolonged exposure of the immunoblot incubated with an anti-GFP antibody (**Figure 4b**). We further validated the usefulness of the pENTR-EUI-based adenoviral vector by infecting HaCaT cells, which are refractory to transfection by a nonviral vector, with the same batch of the adenovirus. As expected, HaCaT cells could not be transfected with pEUI(+) using conventional transfection reagents and thus were nonresponsive to tebufenozide treatment (data not shown). On the contrary, the adenoviral vector was delivered well to HaCaT cells in a MOI-dependent manner, which became highly sensitive to tebufenozide (10 μmol/l) treatment (**Figure 4c**). More importantly, and similar to Cos7 cells, infected HaCaT cells did not show any leaky EGFP expression when not treated with tebufenozide (**Figure 4c**).

To gain more precise quantitative data about the activity and leakiness of the newly generated singular adenoviral vector, we subcloned firefly *Luc* into the *Eco*R I site in the MCS of pENTR-EUI for adenovirus production (Ad/EUI-Luc, **Figure 4d**). For comparison, another singular vector (pENTR-Tet, **Supplementary Figure S2**) that produces a different adenovirus (Ad/Tet-Luc) was created with the *Luc* reporter gene responsive to Tet treatment by combining pTet (**Figure 2b**) and pENTR/D-TOPO. The constructed adenoviral vectors were transduced into HaCaT cells. After 6 hours of infection, the cells were stimulated with fresh cell culture media containing 5 μmol/l Teb or 5 μmol/l Tet for 12 hours before Luc activity was measured. As a control, lysate of noninfected HaCaT cells was used to measure basal responsiveness to the Luc substrate in each experiment. While Ad/EUI-Luc-infected cells did not demonstrate any discernible background expression without Teb stimulation regardless of the viral dose, Ad/Tet-Luc showed a high level of leaky transgene expression (**Figure 4d**). It is noteworthy that, whereas background expression from Ad/Tet-Luc was gradually increased in a MOI-dependent manner (1,661-, 3,399-, and 4,431-fold increase over the basal level with a low, medium, and high dose of virus particles, respectively), the amount of leakiness from Ad/EUI-Luc did not follow the MOI gradient (2.18-, 0.83-, and 1.36-fold increase over the basal level with a low, medium, and high dose of virus particles, respectively; **Figure 4d**), which suggested that the leakiness of the transgene from Ad/EUI-Luc was too low to measure in this experiment. Thus, our data suggested that the adenoviral GvEcR-based singular gene switch can be applied selectively to cell lines that require a high transduction efficiency with low or no leaky expression.

### The adenoviral singular gene switch is sensitive to tebufenozide treatment in mammalian tissues

The liver is highly infected by adenovirus type 5 (ref. 28), which makes this tissue an ideal model for testing the applicability of an adenoviral vector. To test whether the pENTR-EUI-based adenoviral vector is applicable *in vivo*, we injected 100 μl of the vector encoding the *EGFP* transgene (10^7^ virus particles per μl) intravenously. After 24 hours, 100 μl of PBS alone or the same volume of tebufenozide (1 mmol/l) dissolved in PBS was infused twice (once per day) into the mouse via intraperitoneal (i.p.) injection. The liver was extracted 1 day after the final i.p. injection for immunostaining with an anti-GFP antibody. Consistent with the cell culture data, the Ad/EUI-EGFP-infected liver became sensitized to intraperitoneally injected tebufenozide, whereas the other control groups did not demonstrate any detectable transgene expression (see **Supplementary Figure S3**).

**Figure 5:**
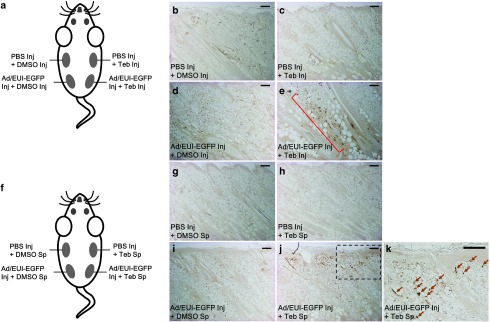
**Local transgene induction in rat dermal tissues by tebufenozide treatment.** In total, 20 μl of adenoviral particles (10^7^ virus particles per μl) was delivered intradermally into rat skin. (**a**) A schematic diagram of *EGFP* transgene activation in rat skin by intradermal tebufenozide injection (Inj). After 24 hours of infection by injecting virus particles prepared in phosphate-buffered saline (PBS) or PBS alone, 20 μl of tebufenozide (100 mmol/l, dissolved in dimethylsulfoxide (DMSO)) or DMSO was administered by intradermal injection and samples were collected 48 hours later. (**b**) A representative tissue sample injected with PBS and DMSO sequentially. (**c**) A representative PBS- and tebufenozide-injected sample. (**d**) A virus-infected sample with subsequent injection of DMSO. (**e**) Cells infected with the adenoviral vector encoding the *EGFP* transgene (Adeno-EGFP) were stimulated by intradermal injection of tebufenozide (brown dots in a red square bracket). **a–e** A single animal was used for the direct comparison of the treatments. (**f**) A schematic drawing of *EGFP* transgene stimulation in rat dermis by the direct spreading (Sp) of 20 μl of tebufenozide (100 mmol/l, dissolved in DMSO) on infected skin. After 24 hours of infection by injecting (Inj) virus particles or PBS, the local infected tissues were treated with tebufenozide or DMSO for 48 hours. (**g**) A representative tissue sample injected with PBS and then treated with DMSO on the skin. (**h**) A sample injected with PBS and then treated with tebufenozide. (**i**) Virus-infected tissue treated with DMSO. **e** EGFP expression was induced in virus-infected dermis by application of tebufenozide to the skin (brown dots). (**k**) A magnified image of the dotted box in (**j**). Arrows represent tebufenozide-responsive cells. **f–k** A single animal was used for the direct comparison of the treatments. Integument is positioned at the top of the images. Teb, tebufenozide. Scale bar = 200 μm.

For direct comparison, we tested whether our adenoviral gene switch is suitable for local delivery of a transgene into skin, which would allow simultaneous and spatial comparison of each experiment in multiple visible areas by exploiting a single animal. To address this issue, we transduced the validated adenovirus (Ad/EUI-EGFP) capable of expressing the *EGFP* reporter gene under the control of tebufenozide stimuli into rat and mouse skin by intradermal injection (**Figure 5** and **Supplementary Figure S4**). As expected, *EGFP* transgene expression was not stimulated in skin tissues treated with virus particles or tebufenozide alone (**Figure 5c**,**d**,**h**,**i**), similar to the internal controls treated with PBS and dimethylsulfoxide (DMSO) (**Figure 5b**,**g**). On the contrary, dermal tissues infected with the adenovirus for 24 hours were responsive to subsequent treatment with tebufenozide via intradermal injection (**Figure 5e**) or direct application to the skin (**Figure 5j**,**k**). However, EGFP expression was higher in dermal tissue injected with tebufenozide (**Figure 5e**) than in samples treated with tebufenozide via direct application to rodent skin (**Figure 5j**,**k**). In addition, by spreading the inducer on the skin, we could stimulate only the marginal dermis close to the surface (**Figure 5j**,**k** and **Supplementary Figure S4e**), while intradermally injected tebufenozide could also elicit EGFP expression in cells organized in deeper subcutaneous tissues, but failed to stimulate adenovirus-infected cells located in the opposite side of the midsagittal plane (**Figure 5a**,**d**,**e**). The distinct responsiveness of the dermis to the chemical inducer according to whether it was injected or spread on the skin may reflect the limited penetrability of tebufenozide dissolved in DMSO, resulting in the activation of transgenes in cells localized only in the upper layers of skin tissue by direct spreading of tebufenozide on the skin, while cells located in deeper subcutaneous tissues can only be stimulated by intradermal injection of tebufenozide. Collectively, our data showed that the GvEcR-based adenoviral singular vector can be used in mammalian tissues as well as in cultured cells.

## Discussion

The main bottleneck when expressing a gene in cells using commercially available transgene inducible systems such as the Tet system is the requirement to deliver two separate units (driver and effector plasmids) into a target cell, which often renders unequal expression of the two units, leading to inconsistent induction by the inducer. Therefore, spatiotemporally controlled transgene expression by a well-orchestrated single vector module without leakiness has long been desired in this field of study.

Besides the Tet system, several types of nonviral and adenoviral gene inducible systems that are responsive to various drugs (*i.e.*, mifepristone or rapamycin) have been developed for use in *in vitro* and *in vivo* applications.^29,30,31,32,33^ Mifepristone, a synthetic steroid that acts as a progesterone antagonist, is commonly used as a transcriptional stimulator for transgene induction,^33^ and the mifepristone-dependent nonviral and viral gene inducible cassettes including all-in-one single vector systems^31,34^ are now available in biological research, although there are contradictory results regarding the background levels of transgenes. Whereas Szymanski *et al*.^34^ argued that their nonviral and adeno-associated viral singular vectors induced low level background expression upon mifepristone stimulation, Maddalena *et al*.^35^ showed that the singular adeno-associated viral gene induction system might not be suitable for *in vivo* transgene induction due to its significant level of leakiness. This discrepancy might be due to the different promoters they employed and/or the discrete promoter configurations. A head-to-head promoter orientation often elicits strong background expression,^34^ which was also adopted by Kügler's group to generate a gene induction cassette with a single vector.^35^ Rapamycin can be utilized as a conditional dimerizer to heterodimerize separate units that can be reassembled into a highly sensitive transcriptional activator.^30,32,33^ A previous report argued that the binary configuration of adenoviral vectors equipped with the dimerizer system showed the highest transgene activation,^32^ which fitted with almost all the required characteristics for an ideal gene inducible switch. However, while mifepristone and rapamycin have been approved by the FDA for clinical use in humans,^36,37^ there remain substantial considerations about their possible adverse effects on embryonic development.^38,39^ Even low doses of mifepristone might affect the ovarian cycle.^33^ In addition, since the dimerizer system becomes almost irreversible after treatment with rapamycin,^40,41^ the dimerized transcriptional units may continuously stimulate transgene expression until the dimer is completely degraded through cellular processes. Thus, among several different gene inducible systems, the EcR-dependent gene induction cassette may have several potential advantages over the other systems. First, since no vertebrate EcR orthologs exist, ecdysone agonists including tebufenozide are not responsive to endogenous vertebrate nuclear receptors.^42,43^ Second, the reversible feature of ecdysone agonists on their cognate receptor (EcR) with favorable pharmacokinetic features, including predictable dose-response relationships, expedites rapid clearance from serum *in vivo*^42,44^ and it is easy to down-tune the transgene expression level by simple washing.^17^ Third, a number of agonists acting on EcR^15,43,45^ have been developed, and such diversity may help to avoid undesirable complications by choosing an appropriate inducer. However, the initially developed and then modified EcR-dependent gene inducible switch (VgEcR/RXR system) has intrinsic flaws because VgEcR, which is composed of a VP16 activation domain fused to an EcR with an altered DNA-binding specificity, must heterodimerize with RXR, which must be exogenously supplemented at a supraphysiological level to gain full transcriptional activity.^13,42^ The EcR-based gene switch was further improved to generate an adenoviral vector using hybrid EcR, which does not require additional supplementation of RXR.^46^ However, the hybrid EcR-based gene switch remains sensitive to the endogenous level of RXR.^46^

To establish more convenient and reliable EcR-based nonviral and adenoviral gene induction tools, we modified the most recently improved binary gene inducible cassette utilizing GvEcR, which is not affected by the endogenous RXR concentration.^17^ The newly designed nonviral pEUI(+) and adenoviral pENTR-EUI gene inducible switches that we developed are singular vectors equipped with the entire regulatory units required for chemical induction of transcription of a target gene with high sensitivity, reversibility, and exceptional low leaky expression; therefore, these systems could induce gene expression with limited variation in expression levels.

Current clinical gene therapies using viral vectors for gene delivery have focused on the safe transfer of a target gene into cells that lack the functional gene product due to a genetic defect or chronic disease.^26,27,47^ However, nonregulated gene expression under the control of constitutive or tissue-specific promoters might cause hyper- or hypo-expression of a gene of interest,^48,49^ resulting in the failure to rectify the cellular pathological condition. Therefore, the application of the inducible transgene expression system would be beneficial for targeted gene therapy. The adenoviral gene delivery system has been used to treat various diseases including malignant tumors because the virus does not induce an insertion mutation in the genome of host cells.^26,27,47^ Using rodents for the *in vivo* studies, we showed that, among the multiple layers of dermis infected with the adenovirus, cells of dermal connective tissue near the apical surface were sensitive to tebufenozide brushed over the skin surface (**Figure 5j**,**k** and see **Supplementary Figure S4e**), while tissue far from the epidermis could be stimulated by intradermal tebufenozide injection (**Figure 5e**). The marginal responsiveness of the dermal tissue to the overlaid chemical inducer suggested that tebufenozide dissolved in DMSO may penetrate only a limited depth into dermal tissues by simple diffusion. Thus, our data suggest that transient spatiotemporal activation of a transgene in rodent skin could be induced by different means of chemical inducer treatment, namely, applying tebufenozide directly to the skin for the activation of distal dermis or through intradermal injection for the stimulation of proximal tissues. In addition, we also showed that the adenoviral gene switch could be stimulated in the liver by administration of tebufenozide via intraperitoneal injection, without any detectable leakiness (see **Supplementary Figure S3**). Our data emphasized that the GvEcR-based singular gene switch with the ability to induce transgene expression on demand should be particularly useful when spatial and temporal control of gene expression is required with negligible background expression.

A plausible drawback of our inducible adenoviral vector is that the gene delivery system could not be suitable for prolonged gene therapy due to its inability to integrate itself into the host genome. A study by Hirsch *et al.*^50^ showed that transgene expression via an adenoviral vector in damaged rat skin lasted for only about 2 weeks, presumably due to its episomal property and cytotoxicity. Thus, it would be advantageous to generate an inducible adenoviral hybrid vector that can stably integrate a transgene into the host chromosome for the long-term temporal modulation of transgene expression.^51,52^ We also emphasize that a transgene in the pENTR-EUI vector should not be longer than 3.5 kb to allow efficient virus packaging. Furthermore, it is noteworthy that the singular gene switch combining the binary feature of the commercially available Tet system would be least suitable for gene therapy applications due to its uncontrollable leakiness, at least in our experimental conditions (**Figures 2c** and **4d**), although the maximum transgene expression level seems to be higher with the Tet-based singular transgene-stimulating vector than with the GvEcR-based gene switch. The data are similar to a previous report, which argued that the Tet activator system (rtTA) showed a considerable level of leaky transgene expression that could be only marginally activated by doxycycline (~2.5-fold at best). The VgEcR/RXR gene induction system shows lower basal activity but relatively weaker responsiveness to an ecdysone analog than the rtTA system.^42^

Collectively, our adenoviral singular gene switch would be highly useful for the treatment of local tissue disorders by modulating the transcriptional activity of a transgene when spatiotemporal regulation of its expression level is prerequisite for proper gene therapy. The other advantage of our adenoviral gene switch system for targeted gene therapy in multiple cutaneous diseases is the simplicity of transgene induction by the direct spreading of tebufenozide on the skin, without inducing any physical tissue damage. To the best of our knowledge, this is the first attempt to generate nonviral and adenoviral singular gene switches based upon GvEcR, which should be useful for the investigation of physiological and pathophysiological gene functions.

Currently, we are attempting to generate a lentiviral vector-based singular gene switch for much broader application of this system to biological and clinical studies.

## Materials and methods

*pEUI(+) and pTet vector construction.* The pPur vector (Clontech, Mountain View, CA) was used as a template to generate pEUI(+). Initially, the pBR322 replication origin of the pPur vector was replaced with the pUC replication origin for enforced plasmid propagation. The in-frame coding sequence of Gal4-VP16-EcR, after erasing the *Eco*R I recognition site located between the Gal4 and VP16 domains of the original clone,^17^ was inserted into pcDNA3 (Invitrogen, Waltham, MA) between the *Hind* III and *Apa* I restriction enzyme recognition sites using an EZ-Fusion Cloning Kit (Enzynomics, Daejeon, Korea) following the manufacturer's instructions. The cytomegalovirus (CMV) promoter with Gal4-VP16-EcR and a downstream BGH poly(A) signaling sequence (CMV-Gal4-VP16-EcR-BGH poly(A)) from pcDNA3 as well as 500 bp spacer sequences from pCMV-Myc (Clontech) were transferred into the modified pPur vector at the *Nde* I and *Pvu* II sites, respectively, using an EZ-Fusion Cloning Kit and a ligation-independent cloning protocol.^20,21^ A 10×UAS with an E1b minimal promoter was subcloned in the *Hind* III site of the pCS2+ vector. The 10×UAS, E1b minimal promoter, and SV40 poly(A) from pCS2+ were transferred en bloc at the *Eco*R I site of the modified pPur vector pre-equipped with CMV-Gal4-VP16-EcR-BGH poly(A) and the 0.5 kb spacer. After inserting a MCS (**Figure 2b**) in the *Eco*R I site, the vector was designated as pEUI(+). Various reporter genes including in-frame *EGFP*, *Luc*, and *ANKRD13A* were subcloned in the *Eco*R I site of pEUI(+). To generate the pTet vector with pcDNA4/TO and pcDNA6/TR (Invitrogen), a DNA fragment encompassing the pUC origin, ampicillin resistance marker, and 2X TetO_2_ under the control of the CMV promoter with BGH poly(A) generated using pcDNA4/TO as a PCR template was inserted in the *Sal* I site of pcDNA6/TR using an EZ-Fusion Cloning Kit. The PCR-amplified *Luc* reporter gene from the pGL3-Basic vector (Promega, Madison, WI) was subcloned in the *Eco*R V site of pTet.

*pENTR-EUI and pENTR-Tet construction.* Given the limitation of the subcloning capacity of the pENTR/D-TOPO (Invitrogen) entry vector (~7.5 kb), the SV40 promoter sequence with the *PAC* gene, SV40 poly(A), and F1 origin (1,997 bp) placed between the driver and effector cassettes of pEUI(+) was deleted for the generation of pENTR-EUI. Unnecessary sequences encoding the ampicillin resistance gene and pUC origin (2,079 bp) were also deleted. The sequential deletion processes of the unnecessary nucleotides to generate the adenoviral entry vector were initially carried out by PCR with *pfu* DNA polymerase to amplify a DNA fragment of 6,169 bp, omitting 1,997 bp composed of SV40 poly(A) and the F1 origin of pEUI(+), and then the PCR product was itself fused using an EZ-Fusion Cloning Kit (Enzynomics) to generate a vector called mini-pEUI (the sequence information is not shown). The driver and effector sequence (4,090 nucleotides) from mini-pEUI(+) was PCR-amplified using *pfu* DNA polymerase, and then subcloned into the pENTR/D-TOPO vector with Topoisomerase (Invitrogen) to generate pENTR-EUI. An *EGFP* or Luc reporter gene was inserted in the *Eco*R I site of pENTR-EUI using a T4 DNA polymerase-based ligation-independent cloning protocol.^21^ For the generation of the pENTR-Tet entry vector, a complete Tet-responsive module (CMV-TetO-BGH p(A)-CMV-TetR-SV40 p(A)) and sequences from attL1 to attL2 required for fusion with pAd/PL-DEST (Invitrogen) were PCR-amplified using the pTet and pEUI(+) vectors as templates, respectively. These two amplified DNA fragments were fused to generate pENTR-Tet using the ligation-independent cloning protocol.^21^ A *Luc* reporter gene was inserted in the *Pst* I site of pENTR-Tet.

*Preparation of adenoviral vectors.* Replication-incompetent adenoviruses (type 5) were generated using the Virapower adenovirus expression system (Invitrogen) following previous reports.^53,54^ Briefly, site-specific DNA recombination between the entry vector (pENTR-EUI or pENTR-Tet, **Figure 4a** and **Supplementary Figure S2**) and the adenoviral destination vector pAd/PL-DEST (Invitrogen) was carried out using LR clonase (Invitrogen). The constructed adenoviral expression vector was transfected into 293A cells using Lipofectamine 2000 (Invitrogen). Cells were grown until 80% cytopathic effect was observed and then harvested to prepare recombinant adenovirus. Adenoviruses were amplified in 293A cells and purified using an Adeno-XTM virus purification kit (Clontech).

*Cell culture and transfection.* HEK293T, Cos7, and HaCaT cells were grown in Dulbecco's Modified Eagle's medium (DMEM) supplemented with 5–10% FBS and antibiotics. HEK293T cells were transfected using the X-tremeGENE 9 DNA transfection reagent (Roche). After 24 hours, the transfectants were treated with various concentrations of tebufenozide (Fluka) and for various amounts of time. The cells were directly observed under a fluorescence microscope or harvested for western blotting.

*Establishment of stable HEK293 tebufenozide inducible cell lines.* The pEUI(+)-ANKRD13A or pEUI(+) plasmid containing a puromycin selectable marker was stably transfected into HEK293 cells using Lipofectamine 2000 (Invitrogen) according to the manufacturer's instructions. Following transfection, cells were selected with 3 μg/ml puromycin (Invitrogen) for 3–4 weeks and maintained in Dulbecco's modified essential medium (DMEM) supplemented with 10% FBS, 100 U/ml penicillin, 100 μg/ml streptomycin, and 1 μg/ml puromycin using cloning cylinders. Of these, 20 independent clones were randomly selected to test inducible expression of Flag-tagged ANKRD13A in the absence or presence of Teb (10 μmol/l) by western blot analysis. One of these clones showed substantial expression and was chosen for the further experiments. Cell lines established with colonies from pEUI(+) mock vector transfection were utilized as experimental controls. The copy number of the pEUI(+)-ANKRD13A transgene in #293-13A was determined to be ≤ 2 by absolute quantitative real-time PCR.^55^

*Western blotting.* Cells were lysed using ProEX CETi Lysis Buffer (TransLab, Daejeon, Korea) with ProEX protease inhibitor cocktail (TransLab) following the manufacturer's instructions. After separation by sodium dodecyl sulfate-polyacrylamide gel electrophoresis (SDS-PAGE) and transfer onto BioTrace nitrocellulose membrane (Poll), the membrane was immersed in 4% skimmed milk dissolved in Tris-buffered saline with Tween 20 (TBST) (10 mmol/l Tris-Cl, 150 mmol/l NaCl, and 0.05% Tween 20, pH 8.0), incubated with diluted primary antibodies for 1 hour, and then incubated with horse radish peroxide (HRP)-conjugated secondary antibodies for another 1 hour. After extensive washing with TBST, the membrane was exposed to ProNA ECL Ottimo (TransLab) substrate. The primary antibodies were purchased from Sigma (mouse Flag M2, F3165), Santa Cruz Biotechnology (GFP, sc-8334; actin, sc-1615), Abcam (ANKRD13A, ab103364), and Calbiochem (α-tubulin, CP06).

*Luc assay.* The *Luc* reporter gene was inserted in the *Eco*R I site of pEUI(+) and the *Eco*R V site of the pTet vector. Luc assays were carried out using the Dual-Luciferase Assay System (Promega). After 24 hours of transfection, cells were stimulated with Tet or tebufenozide at the indicated concentration for 12 hours and then lysed in Passive Lysis Buffer (Promega). Each Luc activity was measured three times, and statistical significance was measured by the Student's *t*-test.

*Reverse transcripase-qPCR.* Total RNA was purified using TRI reagent solution (Ambion). cDNA synthesized with M-MLV reverse transcriptase (ELPIS-Biotech, Daejeon, Korea) and oligo-dT from 1 μg of purified total RNA was subjected to qPCR using Applied Biosystems Power SYBR Green PCR Master Mix (Life Technologies) with primer sets specific for *ANKRD13A* and *GAPDH*. The following primer sets were used to detect target gene expression: *ANKRD13A* forward primer, 5′-GCTGCAGGGCCAGAATGT-3′; *ANKRD13A* reverse primer, 5′-CAGCAAGATGCAATAATGTTCGA-3′; *GAPDH* forward primer, 5′-ATGGAAATCCCATCACCATCTT-3′; and *GAPDH* reverse primer, 5′- CGCCCCACTTGATTTTGG-3′.

*Intradermal injection of adenovirus in rodent skin.* Female Sprague Dawley rats aged 6–8 weeks or C57/BL6 mice aged 7 weeks were used (Orient Bio, Seongnam, Korea). In total, 20 μl of recombinant virus solution (10^7^ particles per μl) prepared in PBS was intradermally injected into the dorsal skin of the animals using a 28-gauge hypodermic needle. After infection for 24 hours, virus-infected skin was stimulated with 20 μl of tebufenozide (100 mmol/l, dissolved in DMSO) by spreading the solution directly on the skin surface or by intradermal injection. For control experiments, the same animal was treated with the same volume of PBS and DMSO on another region of dorsal skin. The animals were sacrificed 2 days after treatment with tebufenozide or DMSO alone, and dorsal skin was carefully removed for immunohistochemical staining. The animal studies were approved by the appropriate institutional review board (the approval number is CNU-00793).

*Adenoviral infection in rodent liver.* Female BALB/c mice aged 8 weeks were used (Orient Bio). In total, 100 μl of vehicle (PBS) or recombinant virus solution (10^7^ virus particles per μl) was intravenously injected using a 28-gauge hypodermic needle. After 24 hours of infection, 100 μl of PBS or the same volume of tebufenozide dissolved in PBS (1 mmol/l) was delivered twice (once per day) into the mouse via i.p. injection. The animals were raised for another 24 hours, and then their livers were removed for immunohistochemical staining.

*Immunohistochemistry.* Prepared skin and liver samples were fixed in 10% formalin for 12 hours before being embedded in paraffin for sectioning. Tissue specimens with a thickness of 4 μmol/l were dewaxed, rehydrated, and then washed three times with PBS. After treatment with proteinase K (1 mg/ml) for 5 minutes at 37°C, the specimens were immersed in H_2_O_2_ for 10 minutes at room temperature before blocking with blocking reagent (Dako, Carpinteria, CA) for 20 minutes. Samples were incubated with an anti-GFP antibody (Santa Cruz Biotechnology, sc-8334) for 1 hour, followed by incubation with a peroxidase-conjugated secondary antibody (Upstate, Lake Placid, NY) for 1 hour. EGFP expression was visualized using a ChemMate DAKO EnVision Detection Kit (DaKo).

**SUPPLEMENTARY MATERIAL** **Figure S1.** Calculation of the transgene copy number in the stable HEK293 cell line that harbors pEUI(+)-ANKRD13A. **Figure S2.** A schematic diagram of the pENTR-Tet vector. **Figure S3.** Transgene induction in the liver by treatment with tebufenozide. **Figure S4.** Local transgene expression in mouse dermal tissues by tebufenozide treatment. Supplementary Materials and Methods
