# Supplementary material for: Ecdysone Receptor-based Singular Gene Switches for Regulated Transgene Expression in Cells and Adult Rodent Tissues
Source: Mol Ther Nucleic Acids. 2016 Sep 27;5(9):e367–. doi: 10.1038/mtna.2016.74 (PMC5056996; doi:10.1038/mtna.2016.74)

**Supplementary Figure S1 Calculation of the transgene copy number in the stable HEK293 cell line that harbors pEUI(+)-ANKRD13A.** (**a**) A primer pair specifically targeting *EcR* was validated by genomic PCR. Genomic RCR of *EcR* produced a single band of 123 bp in the stable cell line and control plasmid samples, but not in non-transfected HEK293 cells. The following primers were used to detect the *EcR* amplicon: forward primer, 5’-AGCATGCGATATTTGCCGAC-3’; and reverse primer, 5’-GTCAGCGGAGACCTTTTGGT-3’. (**b**) Optimization reactions were performed to determine an appropriate concentration using serially diluted (1:2) known quantities of the control plasmid (pEUI(+)-ANKRD13A, top panel) and genomic DNA (bottom panel) extracted from #293-13A by PCR amplification**.** (**c**) The copy number of incorporated pEUI(+)-ANKRD13A in #293-13A was quantified by absolute quantitative real-time PCR (qPCR) analysis. A standard curve of known plasmid copy numbers was created using 2-fold dilutions of plasmid DNA (9.9 kb pEUI(+)-ANKRD13A) by qPCR. The *EcR* transgene was amplified from genomic DNA of #293-13A. The deduced copy number of the transgene in #293-13A calculated from the standard curve is ≤2.

**Supplementary Figure S2 A schematic diagram of the pENTR-Tet vector.** The pENTR-Tet entry vector was created by combining a fragment of pTet (**Fig. 2b**) encompassing all the required Tet-responsive elements with pENTR™/D-TOPO (Invitrogen).

**Supplementary Figure S3** **Transgene induction in the liver by treatment with tebufenozide.** Immunohistochemistry with an antibody against EGFP transgene products in mouse liver. (**a**) A representative liver sample of an untreated mouse. (**b–d**) At 24 h after intravenous (i.v.) injection of PBS or purified Adeno-EGFP virus particles (Ad/EUI-EGFP, 10^9^ virus particles), 100 µl of PBS or the same volume of tebufenozide diluted in PBS (1 mM) was administered twice (once per day) via intraperitoneal (i.p.) injection. The liver tissues were prepared 1 day after the final i.p. injection of PBS or tebufenozide. (**b**) There was no immunostaining upon i.p. injection of tebufenozide following i.v. injection of PBS, similar to the control. (**c**) Ad/EUI-EGFP virus particles did not elicit transgene expression in the liver upon i.p. injection of PBS. (**d**) Treatment with tebufenozide stimulated EGFP transgene expression in the liver infected with the adenoviral vector. Abbreviations: Con, control; Teb, tebufenozide. Scale bar, 100 μm.

**Supplementary Figure S4** **Local transgene expression in mouse dermal tissues by tebufenozide treatment.** In total, 20 μl of adenoviral particles (10^7^ virus particles per μl) encoding the *EGFP* transgene (Ad/EGFP) was intradermally delivered into mouse skin. A single mouse was used for the direct comparison of the treatments. (**a**) A schematic diagram of the stimulation of *EGFP* transgene expression in mouse skin by direct spreading (Sp) of 20 μl of tebufenozide (100 mM, dissolved in DMSO) on virus-infected skin. After 24 h of infection with adenoviral particles prepared in PBS solution or treatment with PBS alone, tissues were stimulated with tebufenozide or DMSO for 48 h. (**b**) A representative tissue sample injected with PBS and then treated with DMSO on the skin. (**c**) PBS-injected tissue subsequently treated with tebufenozide. (**d**) Adenovirus-infected tissue treated with DMSO. (**e**) EGFP expression was induced in dermal tissues infected with the adenovirus upon the application of tebufenozide to mouse skin (brown dots highlighted by arrowheads). Integument is positioned at the top of the images. Abbreviations: Teb, tebufenozide. Scale bar, 200 μm.

**Supplementary Materials and Methods**

*Preparation of genomic DNA for absolute quantitative real-time PCR.* For assays to determine the copy number, genomic DNA was isolated from cell lines by overnight digestion in proteinase K buffer (10 mM Tris-HCl [pH 8.0], 100 mM NaCl, 10 mM EDTA [pH 8.0], 0.5% SDS, and 0.1 mg/ml proteinase K) and phenol-chloroform extraction. To generate a standard curve of the transgene, pEUI(+)-ANKRD13A plasmid DNA was purified from *E. coli* using the QIAGEN Plasmid Kit following the manufacturer’s instructions. The concentration of DNA was determined by measuring UV absorption at 260 nm. Real-time PCR was performed in triplicate in a 20 μl reaction using Power SYBR™ Green Master Mix and the **StepOnePlus™ System (both from** Applied Biosystems) with the following amplification parameters: 10 min at 95°C, followed by 40 cycles of 15 s at 95°C and 1 min at 60°C.

Supplementary Fig. S1.


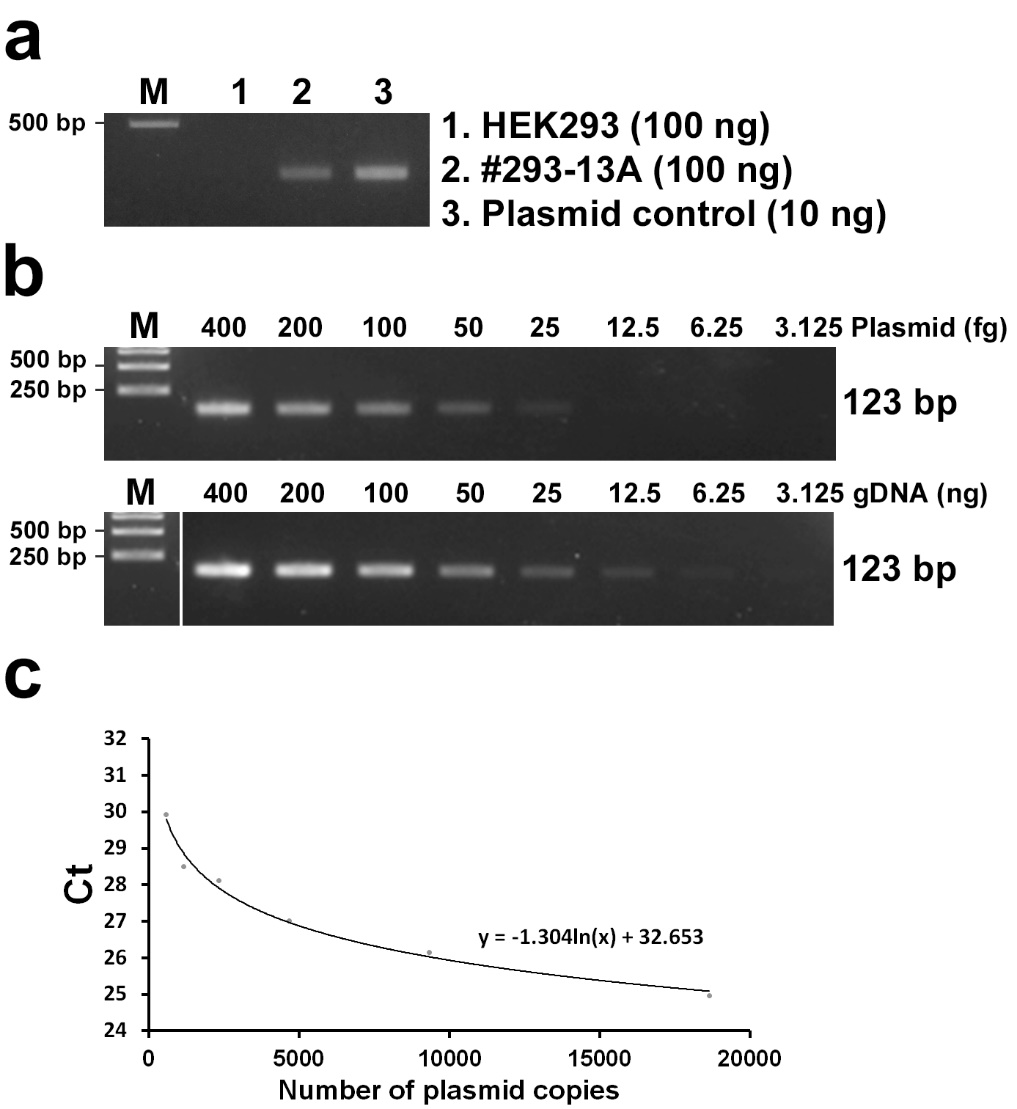


Supplementary Fig. S2.


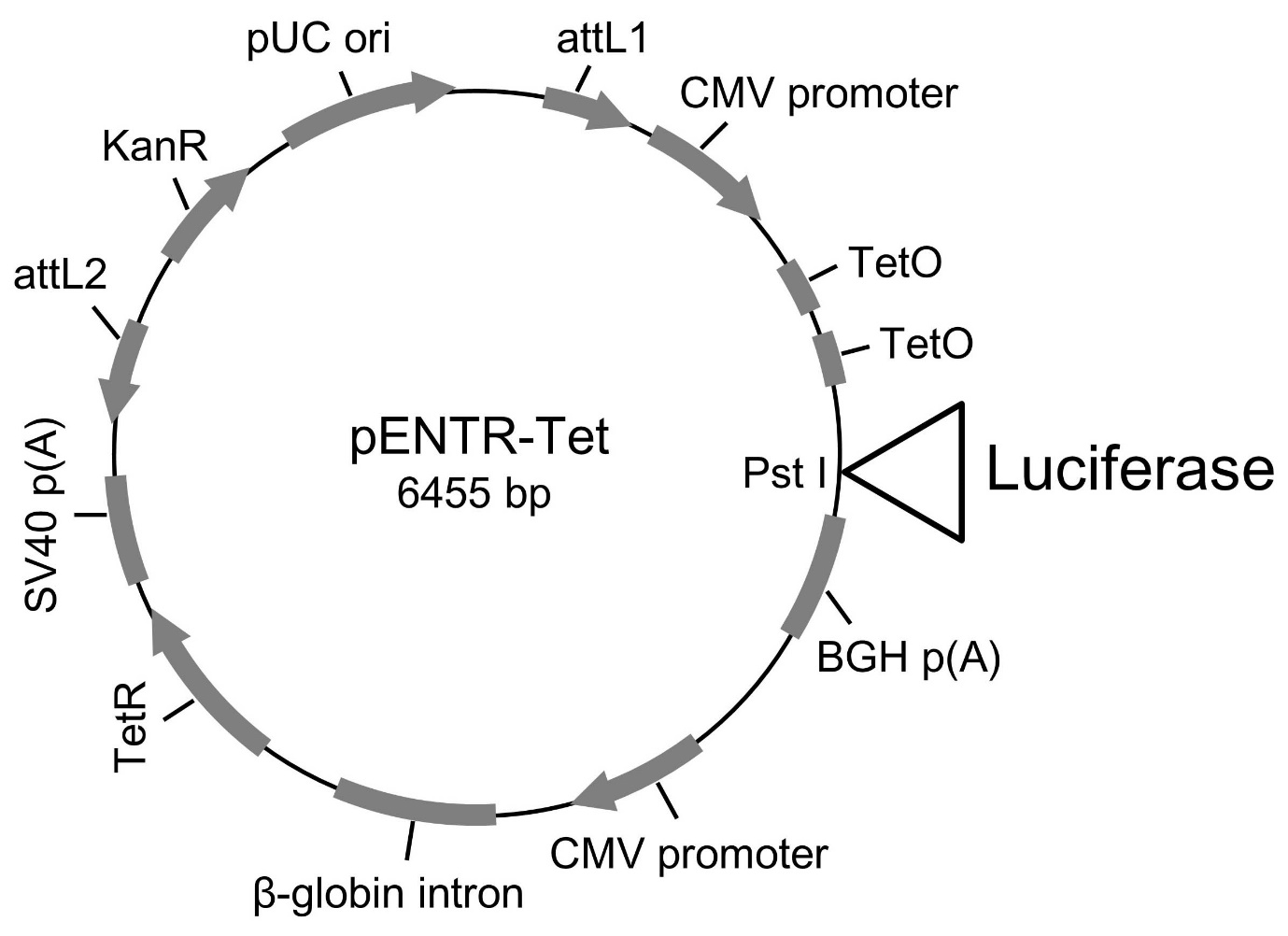


Supplementary Fig. S3.


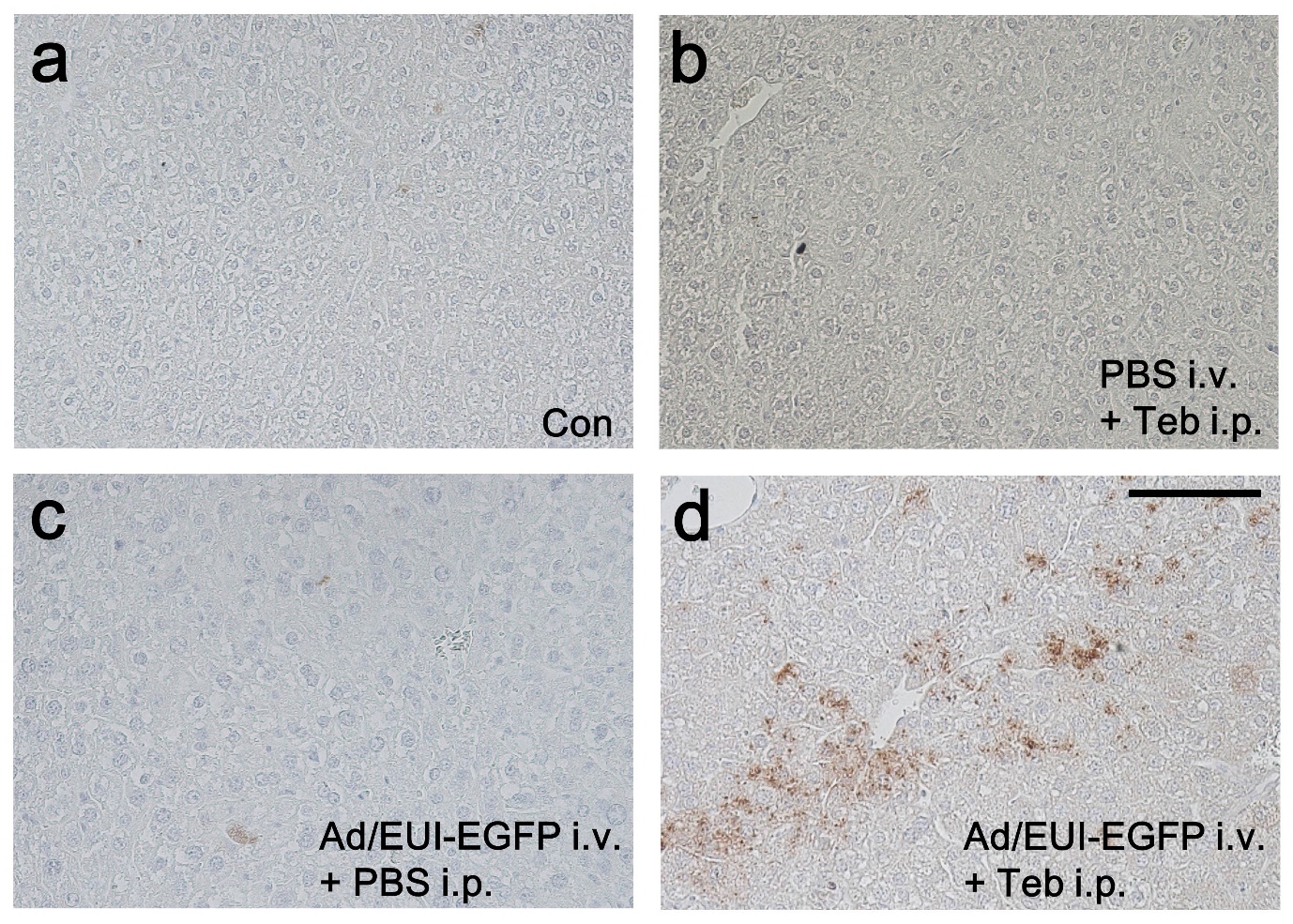


Supplementary Fig. S4.


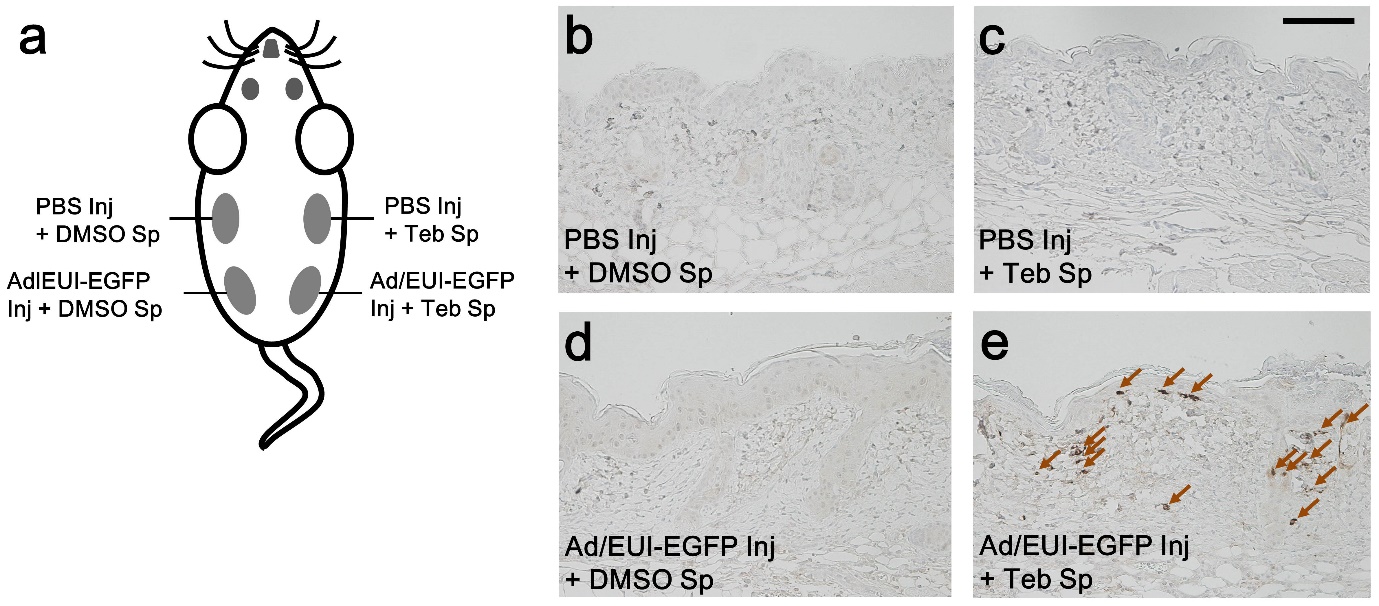

Supplement: Supplementary Figures [file mtna201674x1.doc]
